# Based on the MaxEnt model the analysis of influencing factors and simulation of potential risk areas of human infection with avian influenza A (H7N9) in China

**DOI:** 10.3389/fcimb.2024.1496991

**Published:** 2025-01-03

**Authors:** Zhao Yang, Zhong Da Ren, Jie Wang, Wen Dong

**Affiliations:** ^1^ Faculty of Geography, Yunnan Normal University, Kunming, China; ^2^ Geographic Information System Technology Engineering Research Centre for West-China Resources and Environment of Educational Ministry, Yunnan Normal University, Kunming, China; ^3^ State Key Laboratory of Estuarine and Coastal Research, East China Normal University, Shanghai, China; ^4^ Department of Geography, University College Cork, Cork, Ireland; ^5^ School of Big Data and Information Industry, Chongqing City Management College, Chongqing, China

**Keywords:** H7N9, Maxent model, Influencing factors, risk simulation, China

## Abstract

Exposure to infected animals and their contaminated environments may be the primary cause of human infection with the H7N9 avian influenza virus. However, the transmission characteristics and specific role of various influencing factors in the spread of the epidemic are not clearly understood. Therefore, it is of great significance for scientific research and practical application to explore the influencing factors related to the epidemic. Based on the data of relevant influencing factors and case sample points, this study used the MaxEnt model to test the correlation between human infection with H7N9 avian influenza and influencing factors in China from 2013 to 2017, and scientifically simulated and evaluated the potential risk areas of human infection with H7N9 avian influenza in China. The simulation results show that the epidemic risk is increasing year by year, and the eastern and southeastern coasts have always been high-risk areas. After verification, the model simulation results are generally consistent with the actual outbreak of the epidemic. Population density was the main influencing factor of the epidemic, and the secondary influencing factors included vegetation coverage, precipitation, altitude, poultry slaughter, production value, and temperature. The study revealed the spatial distribution and diffusion rules of the H7N9 epidemic and clarified the key influencing factors. In the future, more variables need to be included to improve the model and provide more accurate support for prevention and control strategies.

## 1 Introduction

The first human case of avian influenza (H7N9) was detected in China in early 2013, followed by identifying the virus in local live poultry markets. At the beginning of the epidemic, the H7N9 avian influenza virus exhibited low pathogenic in poultry. However, the fatality rate of human infection with H7N9 avian influenza virus was significantly higher compared to that of seasonal influenza infection (Zhang et al., 2015). In early 2017, researchers found that a mutant strain was highly pathogenic to poultry and caused multiple outbreaks (Wu et al., 2021; Benmarhnia, 2020; Yu et al., 2014). Studies indicate that live poultry markets may contribute significantly to the transmission of H7N9 avian influenza to humans. This could be attributed to the ideal H7N9 avian influenza virus environment created by the traditional poultry breeding system with a semi-mixed breeding mode (Dong et al., 2015). Meanwhile, poultry farming systems are widespread in the southern coastal areas, recognized as high-risk areas for the spread of avian influenza viruses (Dong et al., 2017; Li et al., 2019; Shan et al., 2019). The outbreak of the H7N9 avian influenza among humans has posed a major threat to both the poultry industry and public health in China. As a result, several cities in southeastern China, including Shanghai, have taken steps to control the outbreak by gradually shutting down all local live poultry markets since April 4, 2013. Although previous studies have investigated the epidemiological features of human H7N9 avian influenza outbreaks and the general risk of disease outbreaks (Chong et al., 2016; Liu and Fang, 2015; Zhuang et al., 2013), no evidence of human-to-human transmission has been found (Bi et al., 2016; Zhou et al., 2018). Studies have found that the outbreak and transmission of H7N9 avian influenza to humans is likely related to poultry trade, vegetation cover, population density, and other factors (Shi et al., 2013; Wu et al., 2015; Zhou et al., 2020). Furthermore, other studies have shown that transmission of human infection with H7N9 avian influenza may be related to climate factors such as temperature, rainfall, and humidity (Fuller et al., 2014; Gao et al., 2020; Hu et al., 2015). In summary, human exposure to infected animals and their contaminated environment may be the primary cause of human infection with the H7N9 avian influenza virus. However, the transmission characteristics of human infection with the H7N9 avian influenza virus and the possible roles of various influencing factors in the transmission process are still unclear (Fang et al., 2013; Kim and Pak, 2019). Therefore, exploring the influencing factors that may be related to the epidemic is an important public health issue to be solved in recent years, which has good scientific research significance and practical application value.

The MaxEnt model is commonly used in biogeography, conservation biology, and ecology. It is used to identify environmental conditions that are related to the occurrence of a particular species and to estimate and predict the distribution of species in a specific region or under particular environmental conditions (Golding and Purse, 2016). Although few studies have utilized the MaxEnt model to assess potential risk areas and related influencing factors of human infection with H7N9 avian influenza, the MaxEnt model has been widely employed in public health research in recent years (Escobar and Craft, 2016; Wardrop et al., 2014). In particular, more and more attention has been paid to infectious diseases related to vector species, such as mosquito-borne diseases and tick-borne diseases (Bui et al., 2017; Liu et al., 2019; Wang et al., 2023), which are to some extent affected by climatic conditions that determine the distribution of vectors (Bui et al., 2017). Based on the MaxEnt model, this study tested the correlation between human infection with H7N9 avian influenza and its influencing factors in China from 2013 to 2017 and conducted scientific simulation and assessment of the potential risk areas for human infection with H7N9 avian influenza in China. The results of the study will help the government and relevant public health departments to formulate targeted epidemic prevention and surveillance strategies.

## 2 Materials and methods

### 2.1 Case data

The data on human infection with H7N9 avian influenza in China were from the Beijing Center for Disease Control and Prevention. From 2013 to 2017, a total of 1,474 people were infected with the H7N9 avian influenza virus in the country, including 155 in 2013, 333 in 2014, 196 in 2015, 265 in 2016, and 591 in 2017.

### 2.2 Poultry breeding data

Poultry breeding data for this study were obtained from the National Bureau of Statistics of China (https://www.stats.gov.cn/), and the poultry slaughter data and Poultry output data for different provinces in the 2013-2017 China Rural Statistical Yearbook were downloaded. In this study, the output value and vector data of poultry in related areas were converted into raster data by ArcGIS.

### 2.3 Meteorological data

The meteorological data in this paper are sourced from the Institute of Tibetan Plateau Research, Chinese Academy of Sciences. The spatial resolution of the data is 0.0083333° (about 1km). This dataset is generated from the global 0.5° meteorological dataset released by CRU and the global high-resolution climate dataset released by WorldClim and downscaled in China using the Delta spatial downscaling scheme. This study is based on the data sets of monthly precipitation, minimum temperature, and maximum temperature during 2013-2017.

### 2.4 Population data

**Figure 1:**
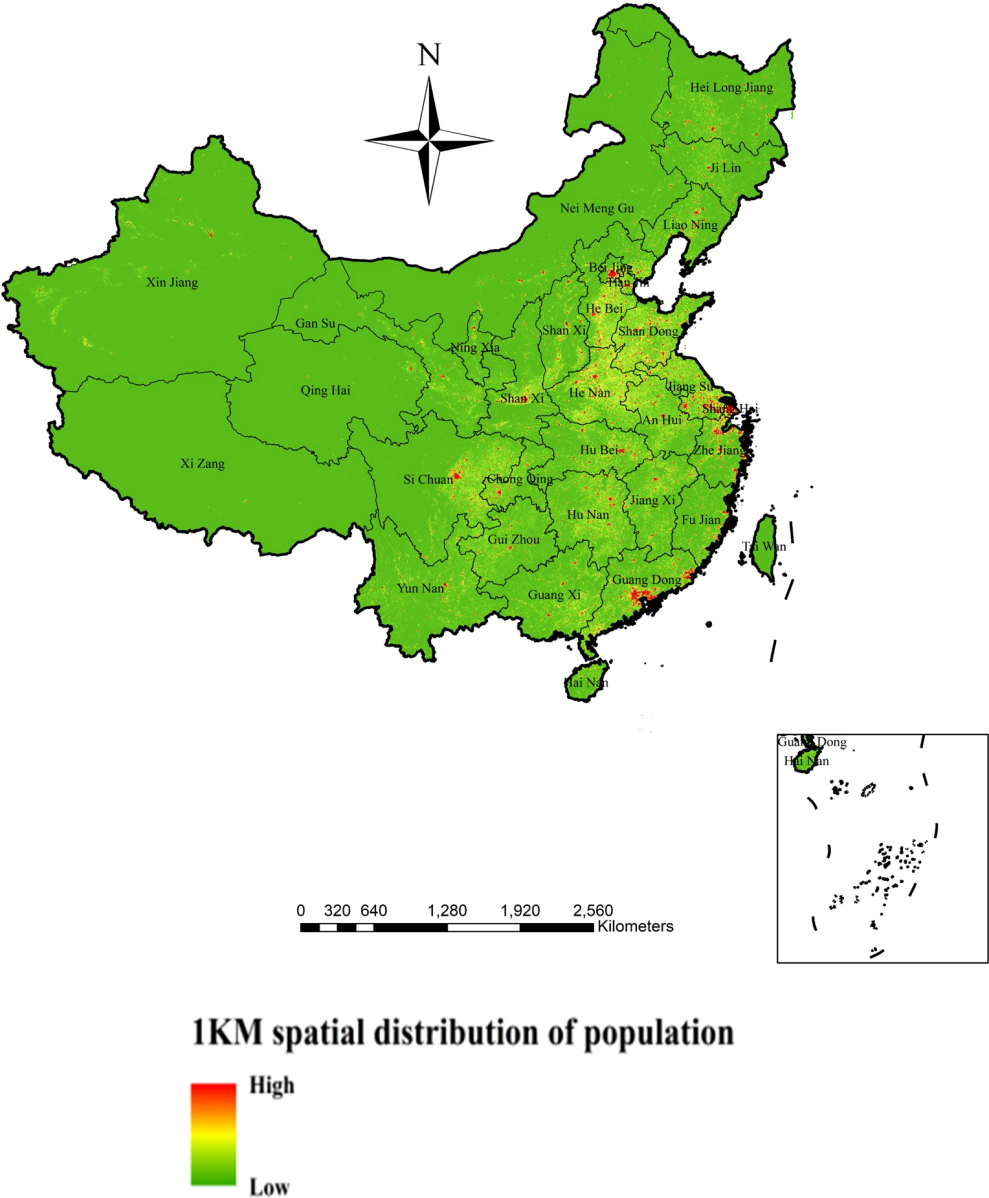
Spatial distribution data of population on a 1km grid.

China’s population distribution is uneven, among which the population density in the southeast is relatively high, and the population density in the northwest is relatively low. In the process of population spatial data processing in China, this study first calculates the population distribution weight of land use type, nightlight data, and settlement density, and then calculates the total weight of each county administrative unit based on the standardized treatment of the influence weights of the above three aspects. Then, based on the grid space calculation, the population quantity on the unit weight is combined with the total weight distribution map. Thus, population data can be spatialized. In this study, the population spatial distribution data of the 1 km grid was finally obtained using the method described above, which better reflects the spatial distribution of the population in China (**Figure 1** Spatial distribution of population). The spatial distribution data of the population obtained in this study are grid data, each grid represents the number of population within the grid range (1 square kilometer), in the unit of person/square kilometer. The data format is gird (http://www.resdc.cn) (Xu, 2017).

### 2.5 Elevation data and vegetation index data

This research USES the elevation data (DEM) comes from us space shuttle endeavor radar topography SRTM (Shuttle Radar Topography Mission) data sets. The data set is a 500m precision data set generated by resampling the latest SRTMV4.1 data (http://www.resdc.cn). In addition, the China Vegetation Index (NDVI) spatial distribution data set used in this paper is SPOT/VEGETATIONNDVI satellite remote sensing data based on continuous time series. This dataset is the annual vegetation index dataset since 1998 generated by the maximum synthesis method (http://www.resdc.cn) (Xu, 2018).

### 2.6 Modeling methods

MaxEnt (version 3.4.1) used in this study is open-source software and can be used for scientific research free of charge (https://biodiversityinformatics.amnh.org/open_source/maxent/). The MaxEnt model can be used to predict the potential geographical distribution of species, and it has been proven to be effective in assessing the potential distribution of ecological or environmental-related diseases (Liu et al., 2018; Artun, 2019; Ma et al., 2019).

**Figure 2:**
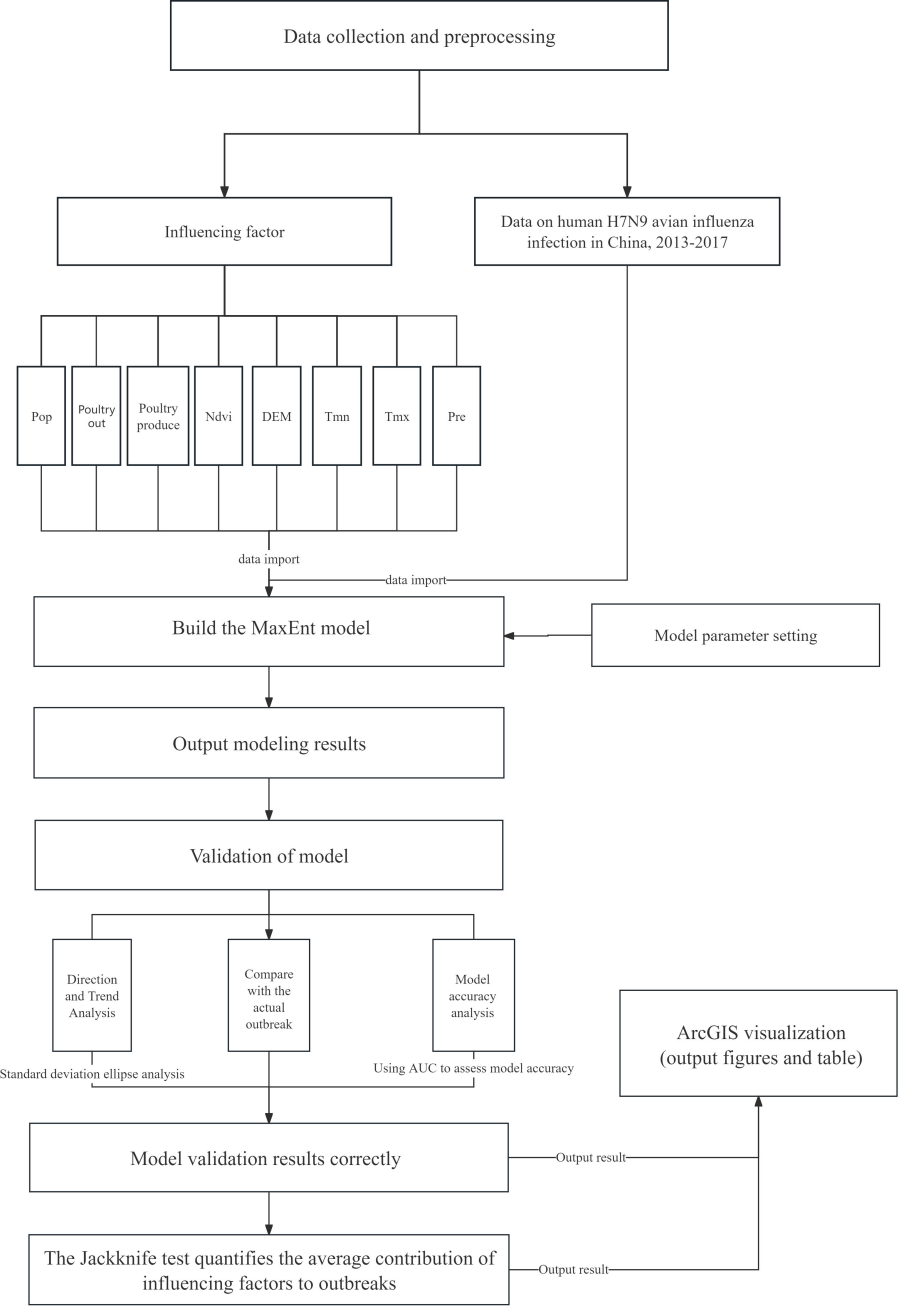
Technical route. In this study, we simulated the potential risk of an outbreak by building a MaxEnt model based on the outbreak data of human infection with the H7N9 avian influenza virus in China from 2013 to 2017 and environmental variables that may be related to the outbreak, including: Pre (average monthly precipitation), Tmx (average monthly maximum temperature), Tmn (average minimum temperature), DEM (altitude), Ndvi (vegetation coverage), Poultry produce (annual poultry).The MaxEnt model was used to simulate the potential risk areas of the outbreak, explore the influencing factors related to the outbreak and assess the risk probability of the outbreak in each area.

Based on relevant influencing factors and case sample points, this study used the MaxEnt model to simulate the potential risk areas of human infection with H7N9 avian influenza in China and assessed the risk probability of epidemic occurrence in relevant areas (**Figure 2** Technical route). In the process of modeling, the maximum entropy principle and jackknife method were used to calculate the influence of each influencing factor to evaluate the impact of different influencing factors on the risk of human infection with H7N9 avian influenza. The data sample points used in this study were the location data of disease points from 2013 to 2017. To reduce the error, 75% of the epidemic data were selected for model training and 25% for model verification (Yuan et al., 2015). To clearly visualize the risk of human infection with H7N9 avian influenza in different regions of China, this study used four levels (Non-risk, low risk, medium risk, high risk) to represent varying degrees of risk. In this paper, the ROC characteristic curve was used to verify the model, and the ROC curve standard was defined as follows: The simulation result of AUC [0.5, 0.6] was “failure”; The simulation results of AUC [0.6, 0.7] were “poor”. The simulation result of AUC [0.7, 0.8] is “average”, the simulation result of AUC [0.8, 0.9] is “good”, and the simulation result of AUC [0.9, 1.0] is “very good” (Ren et al., 2020).

## 3 Results

### 3.1 Simulation results of potential risk areas based on MaxEnt modeling

**Figure 3:**
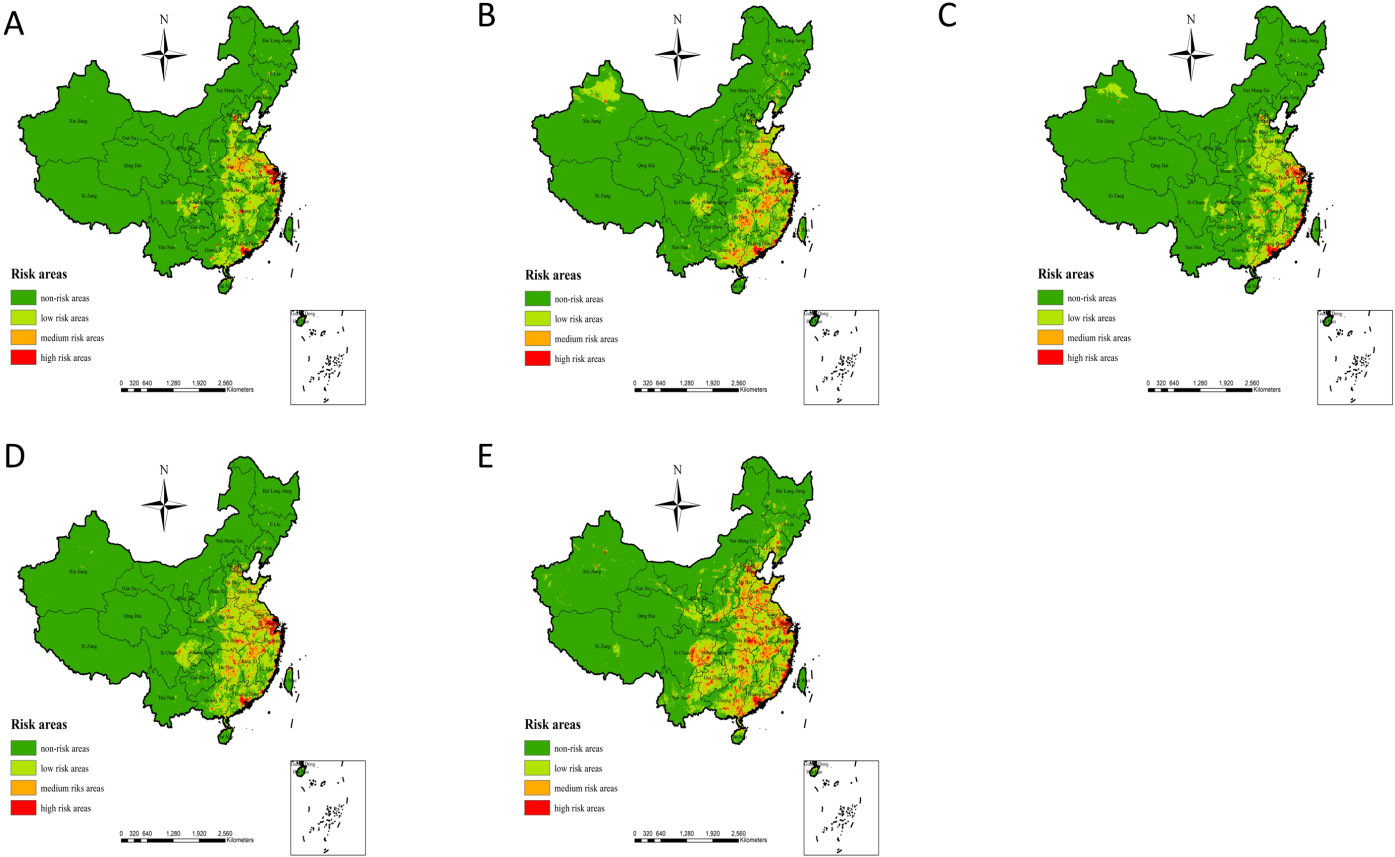
**(A–E)** represent the simulation results of potential risk areas for human infection with H7N9 in 2013, 2014, 2015, 2016 and 2017, respectively avian influenza in China.

The simulation results of potential risk areas for human infection with H7N9 avian influenza in China based on the MaxEnt model show (**Figure 3** Simulation results of potential risk areas). In 2013, the potential risk areas for human infection with H7N9 avian influenza were mainly distributed in Beijing, Hebei, Shandong, Shanxi, Henan, Jiangsu, Shanghai, Anhui, Hunan, Hubei, Zhejiang, Jiangxi, Sichuan, Fujian and other places; In 2014, the potential risk areas for human infection with H7N9 avian influenza were newly added in Xinjiang and some parts of Taiwan, and the risk of epidemic in Shandong, Guangxi and Hunan increased. In 2015, the potential risk areas for human infection with H7N9 avian influenza were mainly Guangdong, Shanghai, Heilongjiang, Xinjiang, Liaoning, Jilin, Sichuan, and other places; The potential risk areas for human infection with H7N9 avian influenza in 2016 indicate that the risk of epidemic in Xinjiang has weakened, while the risk of epidemic in Sichuan, Shandong, Henan, Jiangxi, Hunan and other places has increased. By 2017, the risk of epidemic occurrence was increasing in Guangxi, Guizhou, Sichuan, Heilongjiang, Jilin, Liaoning, Yunnan, Tibet, Qinghai, Gansu, Shaanxi and Inner Mongolia. In general, the risk of human infection with H7N9 avian influenza in China from 2013 to 2017 showed an increasing trend year by year, among which the eastern and southeastern coastal areas of China have always been high-risk areas of the epidemic, and the epidemic risk showed a clear trend of spreading from the eastern and southeastern coastal areas of China to the inland and western regions.

### 3.2 Verification and analysis of model simulation results

#### 3.2.1 Direction and trend analysis

**Figure 4:**
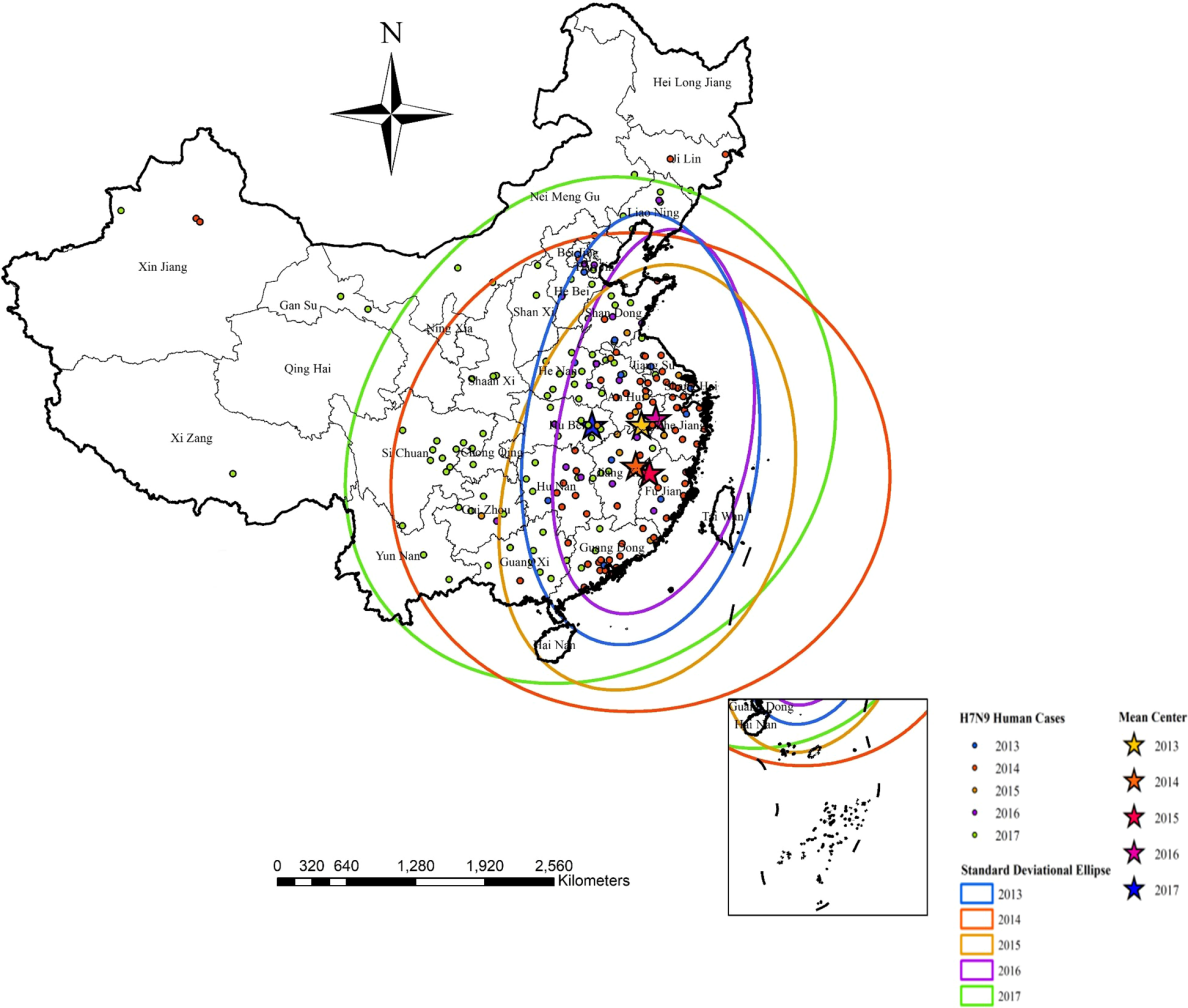
Illustrates clearly the mean centers and the directional trends of influenza A(H7N9) human cases from 2013 to 2017 by standard deviational ellipses.

As can be seen in **Figure 4** (Standard deviational ellipses analysis) during the period 2013-2016, in 2013, the mean center of the ellipse was located in Huangshan City, Anhui Province, and the ellipse was focused in the eastern region of China, mainly including Nanjing, Wuxi, and Suzhou in Jiangsu Province, as well as Chuzhou City, Anhui Province, Huzhou City, Zhejiang Province, and Shanghai City; In 2014, the mean center of the ellipse was focused in Shangrao City, Jiangxi Province, and the ellipse was confined to the eastern and partially central regions of China, mainly including Jiangsu Province, Anhui Province, Zhejiang Province (Hangzhou, Ningbo, and Shaoxing), Guangzhou, Shenzhen City in Guangdong Province, and Shanghai; In 2015, the average center of the ellipse was located in Quzhou City, Fujian Province, and the ellipse was confined to the eastern part of China, mainly including Jiangsu, Anhui, and Zhejiang Provinces (Wenzhou, Jiaxing, Huzhou, Quzhou, and Taizhou), Fujian Province (Fuzhou, Xiamen, Quanzhou, and Zhangzhou), as well as Guangdong Province (Shenzhen, Jiangmen, Meizhou, Shanwei and Dongguan) and Shanghai; In 2016, the mean center of the ellipse was located in Huangshan City, Anhui Province, and the ellipse was concentrated in the eastern part of China, which mainly included Jiangsu Province (Wuxi, Changzhou, and Suzhou), Hefei City, Anhui Province, Zhejiang Province (Hangzhou and Wenzhou), and Shanghai; In 2017, the average center of the ellipse was located in Huangshi City, Hubei Province, and the ellipse was confined to eastern and most of central China, including Jiangsu Province (Suzhou City and Taizhou City), Anhui Province, Zhejiang Province (Ningbo City), Beijing City, Chengde City in Hebei Province, Chengdu City in Sichuan Province and Shanghai City.

#### 3.2.2 Actual outbreaks in 2013-2017

**Figure 5:**
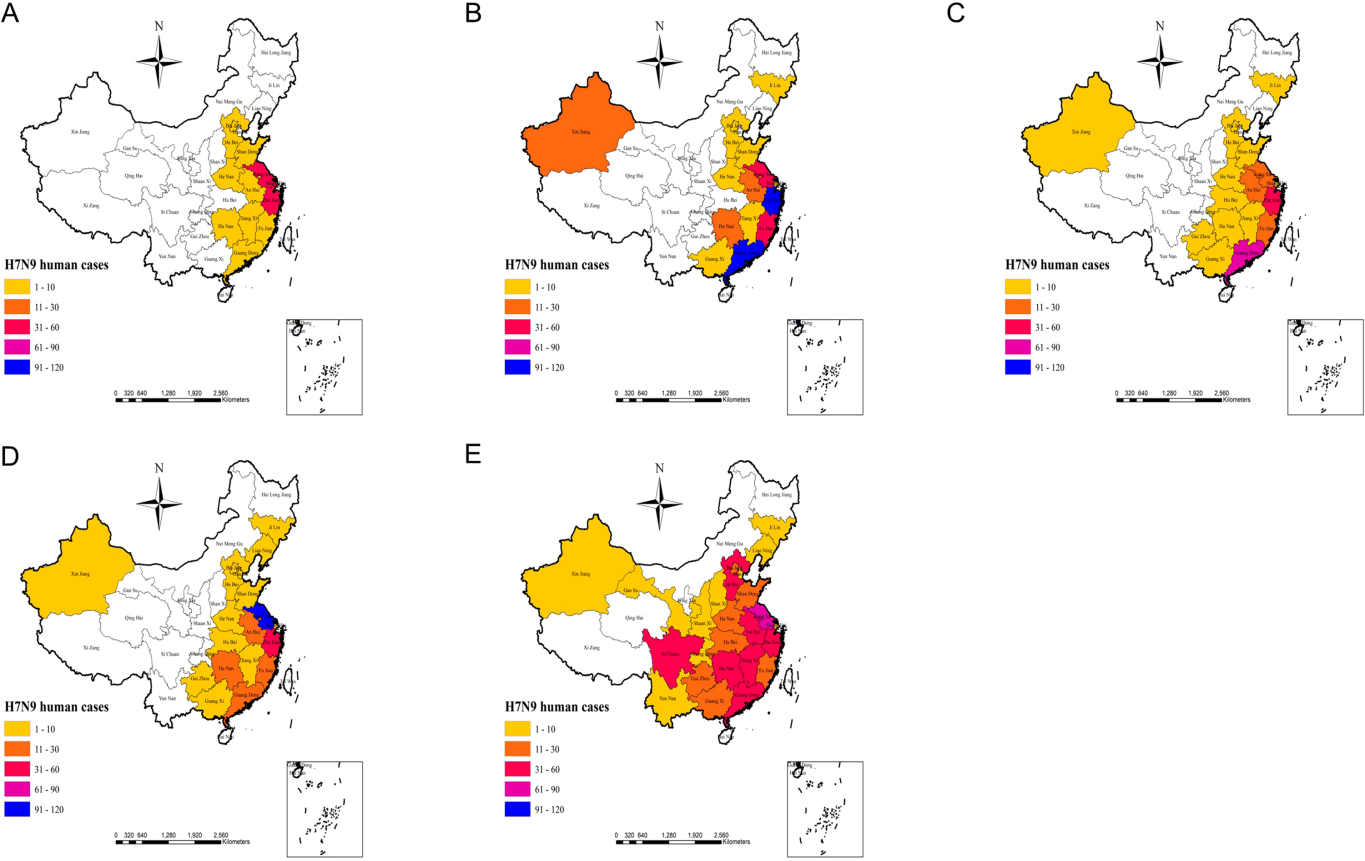
**(A–E)** represents the actual human H7N9 avian influenza outbreaks in China in 2013, 2014, 2015, 2016, and 2017 respectively.

The actual outbreaks of human H7N9 avian influenza outbreaks from 2013 to 2017 (**Figure 5** Actual outbreak situation) demonstrated that, in 2013, Shanghai, Jiangsu, and Zhejiang were hardest hit by the H7N9 avian influenza outbreak with a high number of cases. In 2014, an outbreak of human H7N9 avian influenza occurred in the Xinjiang region, while the Zhejiang and Guangdong regions experienced an increase in cases. In 2015, the human infection with H7N9 avian influenza epidemic in the country exhibited a certain degree of a weakening trend, although the Guangdong region continued to experience the most severe outbreak, and the Zhejiang region also reported an increase in new cases. In 2016, the Jiangsu, Shanghai, and Zhejiang outbreaks were again serious, while the Guangdong region exhibited a certain degree of weakening. In 2017, the Guangxi, Guizhou, Hunan, Jiangxi, Guangdong, Beijing, and Hebei cases increased significantly, while outbreaks also occurred in Yunnan, Sichuan, Shaanxi, Shanxi, Gansu, and Guizhou (**Figure 5** Actual outbreak situation). As can be seen from the results of the modeled risk assessment and the actual outbreaks of human H7N9 avian influenza outbreak risk (**Figure 3** Simulation results of potential risk areas; **Figure 5** Actual outbreak situation), the results of the model assessment and the actual incidence of this study are generally consistent with each other.

#### 3.2.3 Accuracy analysis of epidemic risk model simulation

**Figure 6:**
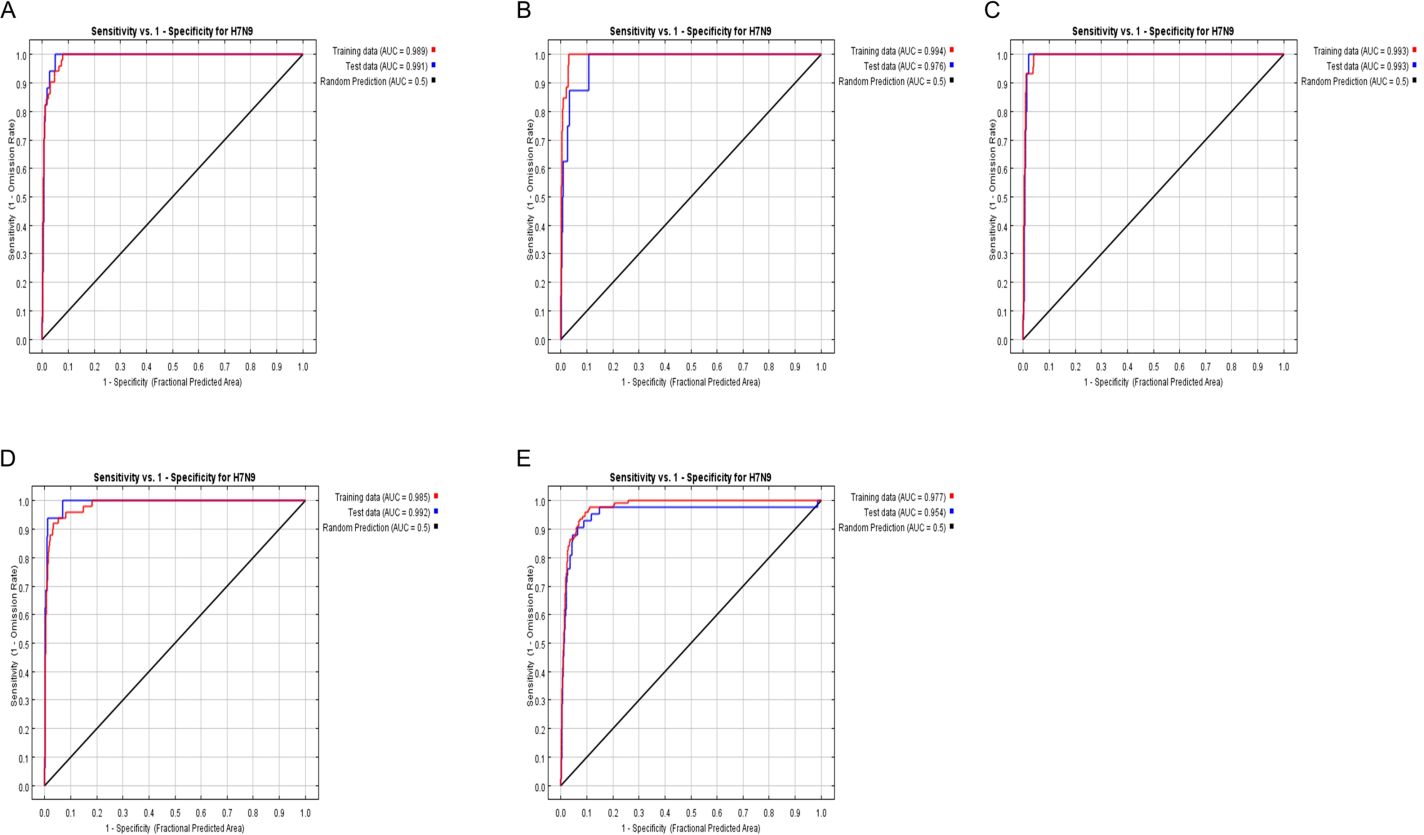
**(A–E)** representing 2013, 2014, 2015, 2016, 2017, the ROC curve and AUC values.

To further verify the risk simulation effect of the model, this study used AUC (area under the ROC curve) to evaluate the model accuracy, where the larger the value of AUC, the higher the accuracy of the model prediction. From **Figure 6** (Analysis of model simulation accuracy), it can be seen that the AUC values of the model training set from 2013 to 2017 are 0.994, 0.989, 0.993, 0.985, and 0.977, respectively, and that of the model test set is 0.976, 0.991, 0.993, 0.992, and 0.954, respectively. It can be seen that the model constructed in this study has relatively good accuracy.

#### 3.2.4 Average contribution of each influencing factor to the outbreak

The contribution rate of each influencing factor to the prediction model of human infection with H7N9 avian influenza is shown in **Table 1**. The results of this study showed that population density was the main influencing factor for the occurrence of H7N9 avian influenza in humans from 2013 to 2017 (72.96% Percent contribution and 34.2 Permutation importance). It was followed by vegetation cover index (8.82% Percent contribution and 14.9 Permutation importance), precipitation(0.91% Percent contribution and 0.92 Permutation importance), altitude (0.64% Percent contribution and 18.88 Permutation importance), poultry slaughter (0.5% Percent contribution and 0.76 Permutation importance) poultry production value (0.42% Percent contribution and 0.84 Permutation importance) and monthly average maximum temperature (0.36% Percent contribution and 1.39 Permutation importance) also had moderate effects on human H7N9 infection in avian influenza. Monthly average minimum temperature (0.13% Percent contribution and 0.08 Permutation importance) had no significant effect on human infection with H7N9 avian influenza (**Table 1**).

**Table 1:** Average contribution rate of each factor to H7N9 avian influenza epidemic from 2013 to 2017.

Variable	Percent contribution (%)	Permutation importance
Tmn	0.13	0.08
Pre	0.91	0.92
Tmx	0.36	1.39
Dem	0.64	18.88
Pop	72.96	34.2
Poultry produce	0.42	0.84
Ndvi	8.82	14.9
Poultry out	0.5	0.76

## 4 Discussion

We used MaxEnt to predict the potential distribution of human H7N9 avian influenza outbreaks in China because the MaxEnt model showed accurate predictive power in data-only simulations and evaluations. Its predictive power is superior to some classical modeling methods, such as random forest and logistic regression models, which generally show accurate predictive power in the simulation and evaluation of only data (Jia and Joyner, 2015). However, it turns out that MaxEnt’s prediction is more accurate when the sample size is smaller, which is more appropriate for human H7N9 bird flu outbreak data (Shcheglovitova and Anderson, 2013; Bean et al., 2012; Wisz et al., 2008). Of course, no model is perfect, and it has its limitations. In order to ensure the accuracy of prediction results as much as possible during the calculation of MaxEnt model, a large number of external parameters need to be input, which leads to large workload in the early stage of data processing and complicated data calculation process. In addition, the accuracy of the model is highly dependent on the quality and integrity of the input data, and insufficient or biased data may lead to inaccurate prediction results (Aloufi, 2024).

Through the standard deviation ellipse analysis, the human infection with H7N9 avian influenza in China showed obvious characteristics of temporal and spatial clustering, and the distribution of cases in some natural years also showed a certain trend of diffusion direction and statistically significant spatio-temporal clustering. The epidemic had the characteristics of overall spread but local clustering. In the analysis, we integrated variables such as temperature, rainfall, altitude, population density, NDVI, annual poultry production, and poultry slaughter to simulate the regional risk distribution of human infection with H7N9 avian influenza in China based on the MaxEnt model.

The model showed that population density was the main influencing factor for human infection with H7N9 avian influenza from 2013 to 2017, followed by vegetation cover index, precipitation, altitude, poultry slaughter, poultry production value, and temperature. The more densely populated areas, the greater the risk of human infection with H7N9 bird flu outbreaks. This result indicates that these areas may be contaminated with the H7N9 avian influenza virus, and cases of H7N9 avian influenza virus infection in humans will likely occur. This may be due to the high mobility of densely populated areas, so people infected with H7N9 avian influenza epidemic. Vegetation coverage also has a greater impact on human infection with H7N9 avian influenza, which may be because areas with high vegetation coverage provide a favorable ecological environment for birds to inhabit, attracting a large number of birds to stay in the area. When the climate conditions in the area are conducive to the survival and reproduction of the H7N9 avian influenza virus, these areas with high vegetation coverage become dangerous sources of infection, thus increasing the probability of outbreaks. Precipitation and monthly maximum average temperature also had some effects on human infection with H7N9 avian influenza, but the monthly minimum temperature had little effect on human infection with H7N9 avian influenza. This may be due to the sensitivity of the H7N9 virus to climate, and the virus can survive for a longer time in a suitable environment.

According to our risk model, starting from Shanghai, high-risk areas for human transmission of H7N9 avian influenza were identified in the southeastern coastal areas and extended to the southwest. Shanghai and much of Guangdong have always been high-risk areas. Shanghai was the site of the first human outbreak of H7N9 avian influenza and still reflects a high risk (Xv et al., 2015). The reason for the outbreak of human H7N9 avian influenza in the eastern coastal areas may be that the local average temperature is very close to the temperature that is most suitable for the survival of avian influenza virus (H7N9); In addition, there are many live poultry processing factories and live poultry farms in these areas, and the traditional live poultry breeding system uses semi-mixed breeding methods, and poultry is the traditional carrier of H7N9 avian influenza virus, creating an ideal environment for the transmission of H7N9 avian influenza virus, which also increases the risk of local residents coming into contact with poultry infected by H7N9 avian influenza virus. Inland, human H7N9 bird flu outbreak risk models show high risk in areas around the Yangtze River Delta, including its major tributaries such as Dongting Lake. These are migratory bird habitats and areas known to be at high risk for human transmission of H7N9 avian influenza (Bui et al., 2017; Liu et al., 2019; Wang et al., 2023). The eastern provinces (including Anhui, Jiangxi, Henan, Shandong, and Hubei) also contain some high-risk areas, but as can be seen from **Figures 1**–**6**, there are relatively few high-risk areas in these provinces. In these provinces, the driest months have very little rainfall, which may also increase the potential for the flu virus to spread from poultry to humans. This reminds us that in the dry season, we should strengthen the prevention of human infection with H7N9 avian influenza, and reduce the risk of poultry transmission of H7N9 avian influenza virus to humans. Vegetation coverage also has an impact on human infection with H7N9 avian influenza, which may be because the higher the vegetation coverage, the more suitable for wild birds to survive, which also provides a good way for the transmission of human infection with H7N9 avian influenza.

In our study, we also found that most high-risk areas from 2013 to 2016 were mainly concentrated in coastal areas such as Shanghai, Guangdong, Zhejiang, and Jiangsu. As of 2017, the epidemic has clearly spread from the eastern coastal areas to the western inland areas, and the outbreak has spread most seriously in 2017. It is worth noting that in 2017, the affected areas began to spread inland from the coastal areas. From 2013 to 2017, the central point of human infection with H7N9 avian influenza was mainly concentrated in Anhui, Fujian, Zhejiang, Jiangxi, and Hubei regions, and showed a trend of continuous westward spread in the eastern coastal areas. This shows that some southeastern coastal provinces and some central provinces have always been high-risk areas for human infection with H7N9 avian influenza, and gradually extending inland. Given this, epidemic prevention departments in these provinces should strengthen epidemic prevention measures to prevent the spread of the epidemic to the inland.

## 5 Conclusion

Based on the MaxEnt model, this study analyzed the spatial potential distribution of human infection with H7N9 avian influenza from 2013 to 2017 and the law of epidemic spread, and explored various influencing factors related to epidemic occurrence. Our research results can provide some auxiliary decision support for prevention work. Although the MaxEnt model provides some useful insights for the study of avian influenza virus, because the transmission of human H7N9 avian influenza virus is a rather complex process, we still need to incorporate more influencing factors into our model and expand the sample size in future work to improve the prediction accuracy and wide applicability of the model.

## Data Availability

The original contributions presented in the study are included in the article/supplementary material. Further inquiries can be directed to the corresponding authors.
